# Surgical Removal of a Tick‐Bite Region Without the Presence of an Insect Body

**DOI:** 10.1002/ccr3.70801

**Published:** 2025-08-15

**Authors:** Tomoaki Takada

**Affiliations:** ^1^ Sumikawa Takada Dermatology Clinic Sapporo Hokkaido Japan

**Keywords:** dermoscopy, mouthparts, pathology, tick bites, tick removal

## Abstract

In the absence of an insect body, the diagnostic dermoscopic features of tick bites in the patient included a puncture hole in the tick‐bite lesion located at the center of an erythematous area, along with linear‐to‐irregular white exfoliating imprints consistent with chelicerae.

## Graphical abstract

Surgical excision of a tick‐bite lesion revealed retained mouthparts despite the absence of a visible tick body. Dermoscopy demonstrated chelicerae imprints, and histopathology confirmed the presence of residual tick structures. This case highlights the combined diagnostic value of dermoscopy and histopathology in cases where the tick body is not found.

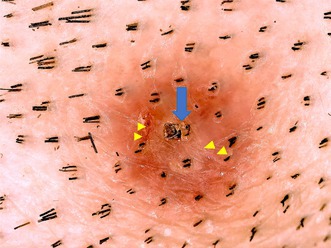

## 1 Introduction

Ticks may harbor pathogens and transmit infections when injecting salivary gland material. The most common viral infections are severe fever with thrombocytopenia syndrome, tick‐borne encephalitis, rickettsial infection, Japanese spotted fever (JSF), and borrelia infection in the form of Lyme disease (LD). Currently, tick attachment is often unconfirmed at the onset of the disease [1, 2, 3].

Typically, only the crust that forms after tick shedding is recognized as the “bite.” Tick bites are not uncommon in dermatological practice and are prevalent in certain regions. However, no standardized guidelines exist for managing patients with tick bites. Therefore, they are addressed according to the experience and judgment of the physician overseeing the patient. Issues arising from this include the absence of evidence backing a specific method for tick removal; variations in judgment regarding tick removal, depending on the physician; ambiguity surrounding the handling of removed ticks (storage methods and requests for identification, among others); the absence of a defined risk assessment for infectious diseases; and the routine administration of antibacterial prophylaxis, which may seem unnecessary [2, 3].

Because spirochete transmission can occur after a minimum of 36 h of attachment, ticks should be promptly removed once discovered. Ticks can be removed using tweezers and stored for later identification. To remove ticks, utilize fine‐tipped tweezers to grasp parts of the mouth as close to the host's skin as possible, and steady traction should be applied directly away from the skin [4].

In one study, surgical removal of the tick from the skin under local anesthesia emerged as the most reliable method of removing ticks in cases of tick bites [3]. However, only a few specific case reports support this finding.

This study reports a case of surgical excision of an occipital tick bite under local anesthesia, where the tick itself had detached before the patient visited the hospital. Clinical, dermoscopic, and histopathological images of the tick‐bite site and the surrounding skin, all as a single mass, were recorded.

## 2 Case History and Examination

**FIGURE 1:**
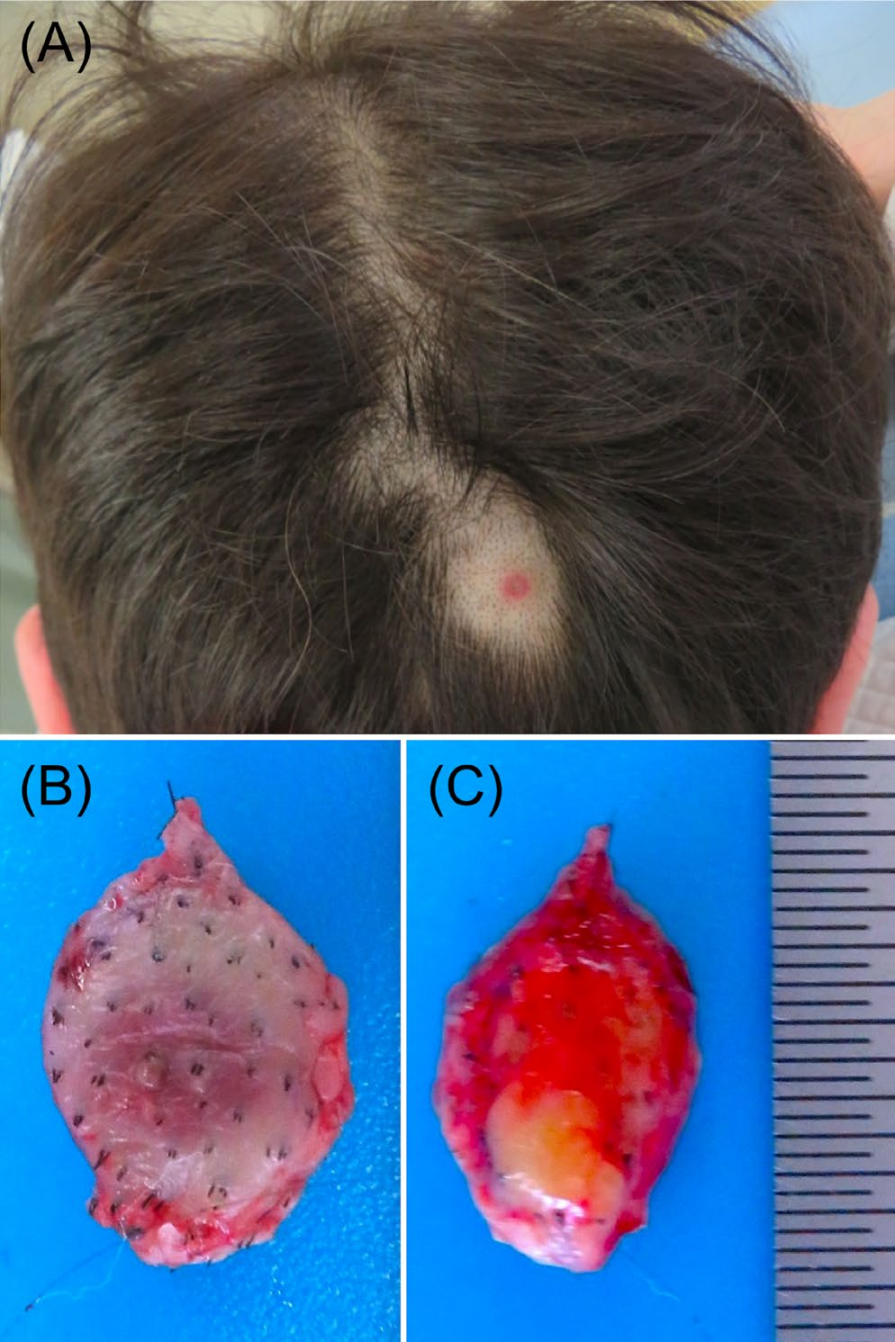
Clinical presentation and surgical resection. (A) An erythema with a central black spot can be seen on the occipital area. (B) Ventral and (C) dorsal views of the resected specimen.

A 66‐year‐old man arrived at our clinic with tick bites on the back of his head that he had sustained 2 days prior. The patient manually removed the attached tick using a commercially available tick‐detachment device. The initial clinical examination revealed erythema with a central black spot in the occipital region (Figure 1A). The tick's body had already been shed and was not found. No mass was palpable in this area.

**FIGURE 2:**
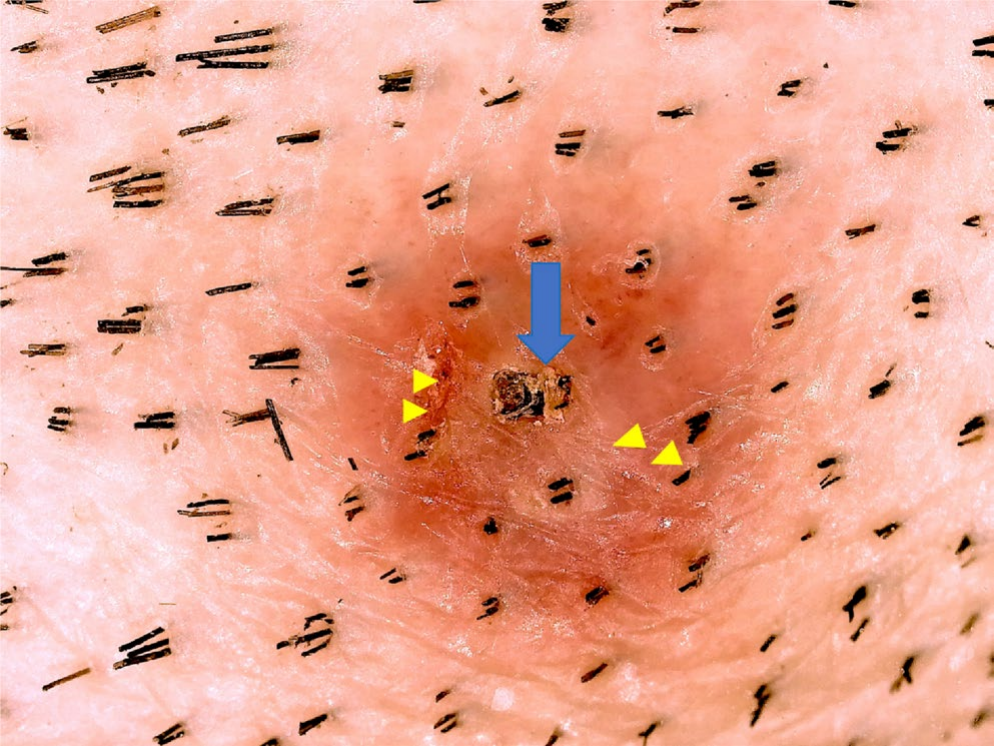
Dermoscopic findings. Dermoscopy shows a homogeneous pattern, milky‐red areas, and a blackish area (indicated by blue arrow) in the center. A red linear‐to‐irregular white exfoliating image (indicated by yellow triangle) by chelicerae is observed. Skin defect opening (namely eschar) is observed bordering two hairs, with a uniform black area inside. Its surface is covered with crust, damaged, and degenerated keratinous material.

Dermoscopy revealed a homogeneous pattern, milky‐red areas, and blackish foreign body‐like structures stuck in the center. Tick bite openings with a uniform black appearance were observed, bordering two hair strands. The surface was covered with damaged and degenerated keratinous material. Linear‐to‐irregular white exfoliating imprints from the tick's chelicerae were also observed (Figure 2).

### 2.1 Differential Diagnosis, Investigation, and Treatment

The differential diagnosis was tick breakage owing to the patient's removal of the attached tick. A simple skin excision, including the erythematous area on the lateral side and the fat layer on the deeper side, was performed for diagnostic and therapeutic purposes (Figure 1B,C).

**FIGURE 3:**
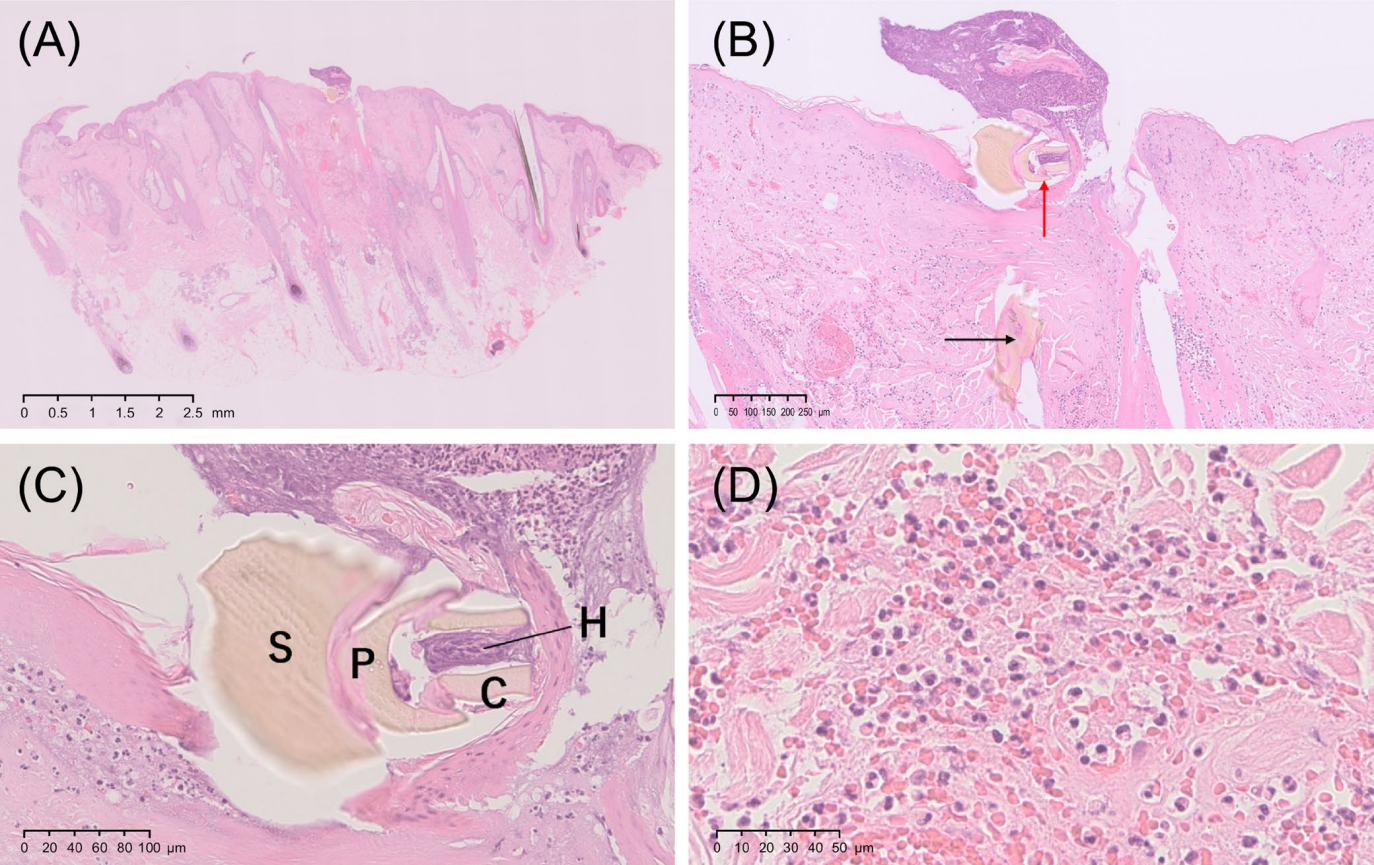
Histopathological findings using hematoxylin and eosin (H&E) staining. (A) Complete image of the resected specimen. Partial or total loss of the epidermis, ulceration, total keratinocyte degeneration, and necrosis can be observed. H&E‐stained specimen; original magnification: ×10; scale bar, 2.5 mm. (B) In the center of the lesion, tick mouthparts (red arrow) can be observed in the uppermost layer of the dermis opening into the epidermal defect. The damaged portion of the tick body was found in a medium‐sized dermal fissure (black arrow). H&E‐stained specimen; original magnification: ×50; scale bar, 250 μm. (C) The tick's mouthparts, the paired chelicerae C ＝ chelicerae and H ＝ hypostome, can be observed between the two P ＝ palps. S ＝ Scutum can be observed on top of the body. H&E‐stained specimen; original magnification: ×200; scale bar, 100 μm. (D) Infiltration of inflammatory cells, including eosinophils and neutrophils, can be observed around blood vessels and between sclerosis of collagen fibers in the dermis, with extravascular exposure of red blood cells. H&E‐stained specimen; original magnification, ×400; scale bar, 50 μm.

Histopathological analysis revealed partial to complete epidermal loss and keratinocyte degeneration (Figure 3A). Tick mouthparts were observed at the center of the lesion in the uppermost dermal layer, opening into the epidermal defect. A damaged part of a tick's body was observed in a medium‐sized dermal fissure (Figure 3B). The remnant mouthparts noted within two palps were chelicerae and a hypostome. The scutum on the top of the tick's body was disconnected (Figure 3C). An infiltration of inflammatory cells, including eosinophils and neutrophils, around blood vessels and between collagen fibers in the dermis, with extravascular exposure of red blood cells, was also observed (Figure 3D).

### 2.2 Outcome and Follow‐Up

The patient had no comorbidities and exhibited no signs of local or systemic complications, including erythema migrans, fever, or neurological symptoms during the 6‐month follow‐up period.

## 3 Discussion

The findings of this study highlight the importance of dermoscopy and histopathology in detecting tick remnants, particularly in cases where visible tick parts are absent. These diagnostic tools significantly improve diagnostic accuracy and support informed clinical decision‐making. Moreover, incorporating surgical excision in high‐risk scenarios has been shown to improve patient outcomes, highlighting the effectiveness of this approach in managing tick bites.

Ticks are known vectors of infectious diseases, such as rickettsioses, including JSF, and borreliosis, such as LD. Since the first report of severe fever with thrombocytopenia syndrome in Japan in 2013, public awareness of tick‐related risks has significantly increased, leading to a rise in clinical consultations for tick‐bite management [3]. If a tick bite occurs, it is advisable to seek medical attention. Some tick species have long suboral structures that anchor firmly to the host with cement‐like material, making them or their remnants difficult to remove. The risk of pathogen transmission increases with incomplete tick removal. Forcibly and unsuccessfully attempting to remove ticks, a practice known as “improper removal technique,” can result in incomplete removal, leading to suppuration and nodule formation. Therefore, ensuring the complete removal of the tick—including surgical intervention when necessary—is crucial [3, 5].

Recent studies suggest that manual extraction using fine‐point tweezers is an effective and accessible method for tick removal [6, 7]. A systematic review conducted in 2017 highlighted the limited evidence supporting commercial tick removal devices over manual methods but emphasized the need for large, high‐quality studies to strengthen evidence‐based recommendations [8]. Public education regarding proper tick removal techniques and associated risks should be prioritized [9].

Surgical excision may be considered in selected cases, such as when a tick is smashed or crushed during a failed removal attempt or when dealing with small nymphs, which are challenging to remove completely. This approach ensures the complete removal of mouthparts, minimizes the risk of secondary infection, and prevents granuloma formation. However, surgical removal may be unsuitable for sensitive areas, such as the eyelids [10]. Updated guidelines recommend prompt tick removal regardless of the time elapsed, emphasizing that delays increase the risk of infection and complications [2, 4].

Clinicians should remain vigilant for signs of tick‐borne diseases, including erythema migrans or systemic symptoms, as delayed diagnosis can lead to significant morbidity. Antibiotic prophylaxis is not routinely recommended but may be considered in endemic areas with high‐risk scenarios, such as prolonged tick attachment (> 72 h) or multiple bites. Ecological information indicates that the local infection rate of ticks with *Borrelia burgdorferi* exceeds 20% [2].

Dermoscopy plays a critical role in diagnosing tick bites, particularly in identifying residual parts or larval forms. In our case, dermoscopy revealed unique findings, such as chelicerae imprints, providing diagnostic clues and highlighting the utility of this tool [11, 12, 13]. The study results highlight the novelty of these findings by presenting concrete dermoscopic evidence, such as chelicerae imprints, that not only corroborate earlier findings but also extend the clinical significance of these diagnostic markers. Notably, these findings address gaps in the literature by demonstrating the effectiveness of histopathological analysis in cases where no visible tick body is observed.

Histopathological analysis further aids in diagnosis, especially in cases with no visible tick remnants. Criteria established by Murasawa and Kimura [14] remain a valuable reference, as demonstrated in our case, where histological findings confirmed the presence of tick remnants despite the absence of a visible tick body. This underscores the importance of integrating clinical, dermoscopic, and histopathological evaluations in tick bite management.

**FIGURE 4:**
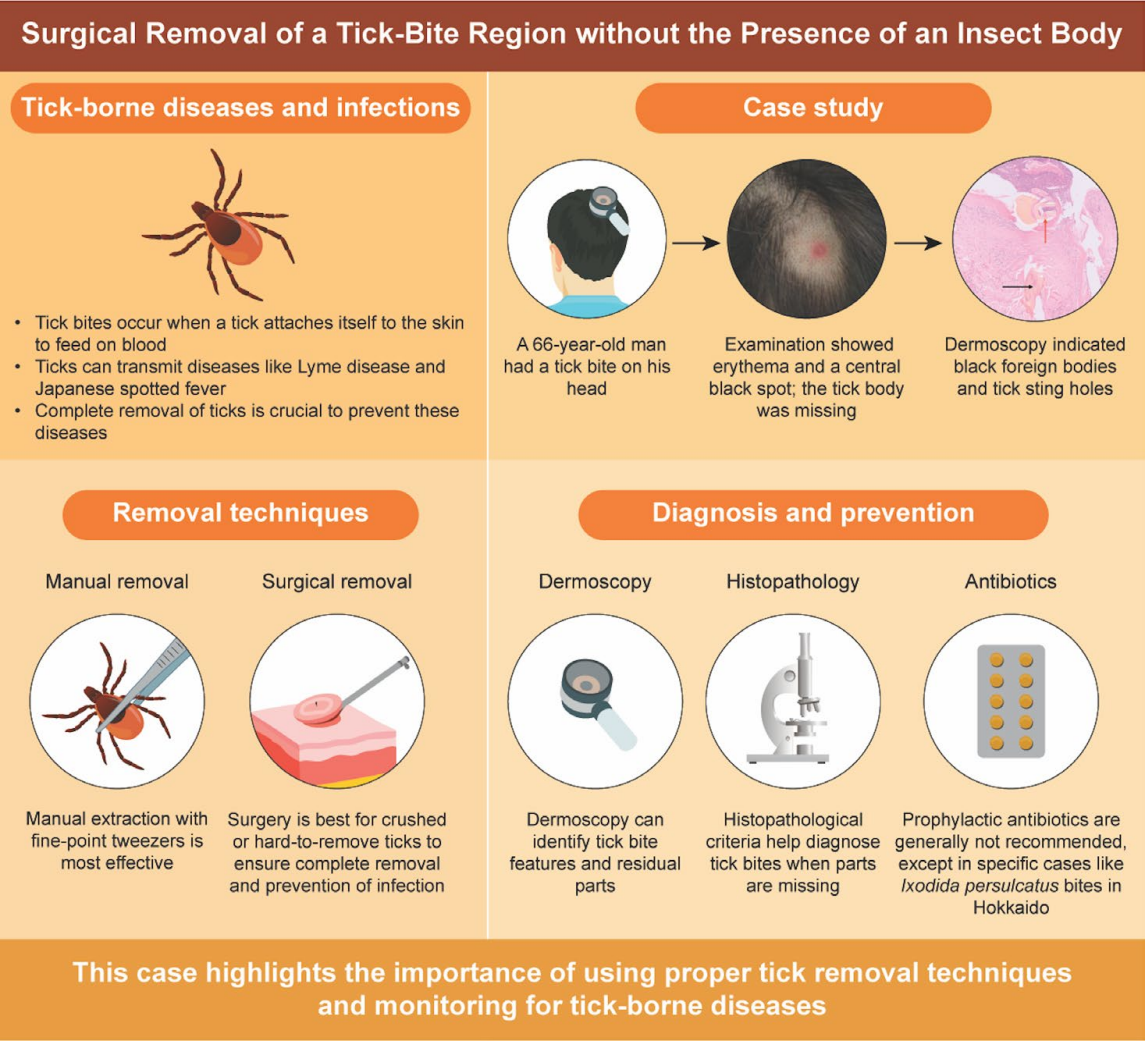
Graphic abstract illustrating the diagnostic workflow for a tick‐bite lesion without a visible tick body. Dermoscopy revealed a central puncture hole with white exfoliating imprints consistent with chelicerae, which were confirmed by histopathology after surgical excision.

In conclusion our case highlights the importance of a comprehensive approach to tick‐bite management, including proper removal techniques, dermoscopic evaluation, and histopathological confirmation. Surgical excision remains the preferred method in cases involving residual parts or high‐risk scenarios. Public awareness campaigns and continued research are crucial for improving the prevention, diagnosis, and treatment of tick‐borne diseases. Moreover, clinicians must educate patients about monitoring for symptoms and the potential need for medical follow‐up, particularly in endemic regions. By incorporating these strategies, healthcare providers can reduce complications and improve outcomes in patients presenting with tick bites (Figure 4).

## 4 Limitations

Limitations of this study include its focus on a single patient, which restricts the generalizability of the findings. Future multicenter studies are crucial to validate the broader applicability of these results and to further refine diagnostic techniques such as dermoscopy and histopathology. Developing standardized guidelines for these approaches will enhance their effectiveness in managing tick bites.

## Author Contributions

**Tomoaki Takada:** conceptualization, data curation, funding acquisition, investigation, methodology, project administration, resources, software, supervision, validation, visualization, writing – original draft, writing – review and editing.

## Ethics Statement

All procedures adopted in this study adhered to the ethical standards of the World Medical Association Declaration of Helsinki. Ethical approval was not required for this study according to local and national guidelines.

## Consent

Written informed consent was obtained from the patient for the publication of this case report and the accompanying images.

## Conflicts of Interest

The author declares no conflicts of interest.

## Data Availability

All data generated or analyzed during this study are included in this article. Further inquiries can be directed to the corresponding author.

## References

[1] S. Madison‐Antenucci , L. D. Kramer , L. L. Gebhardt , and E. Kauffman , “Emerging Tick‐Borne Diseases,” Clinical Microbiology Reviews 33, no. 2 (2020): e00083‐18, 10.1128/CMR.00083-18. 31896541 PMC6941843

[2] T. Benzoni and J. S. Cooper , “Tick Removal,” in StatPearls [Internet] (StatPearls Publishing, 2024), https://www.ncbi.nlm.nih.gov/books/NBK441855/. 28722885

[3] M. Natsuaki , “Tick Bites in Japan,” Journal of Dermatology 48, no. 4 (2021): 423–430, 10.1111/1346-8138.15779. 33586799

[4] P. M. Lantos , J. Rumbaugh , L. K. Bockenstedt , et al., “Clinical Practice Guidelines by the Infectious Diseases Society of America (IDSA). American Academy of Neurology (AAN), and American College of Rheumatology (ACR). 2020 Guidelines for the Prevention, Diagnosis and Treatment of Lyme Disease,” Clinical Infectious Diseases 72, no. 1 (2021): e1–e48, 10.1093/cid/ciaa1215. 33417672

[5] J. Suppan , B. Engel , M. Marchetti‐Deschmann , and S. Nürnberger , “Tick Attachment Cement‐Reviewing the Mysteries of a Biological Skin Plug System,” Biological Reviews of the Cambridge Philosophical Society 93, no. 2 (2018): 1056–1076, 10.1111/brv.12384. 29119723 PMC5947171

[6] S. Roupakias , P. Mitsakou , and A. A. Nimer , “Tick Removal,” Journal of Preventive Medicine and Hygiene 52, no. 1 (2011): 40–44. 21710824

[7] A. A. Akin Belli , E. Dervis , S. Kar , O. Ergonul , and A. Gargili , “Revisiting Detachment Techniques in Human‐Biting Ticks,” Journal of the American Academy of Dermatology 75, no. 2 (2016): 393–397, 10.1016/j.jaad.2016.01.032. 26944595

[8] V. Huygelen , V. Borra , E. De Buck , and P. Vandekerckhove , “Effective Methods for Tick Removal: A Systematic Review,” Journal of Evidence‐Based Medicine 10, no. 3 (2017): 177–188, 10.1111/jebm.12257. 28464468

[9] A. R. Şahin , H. Hakkoymaz , A. M. Taşdoğan , and E. Kireçci , “Evaluation and Comparison of Tick Detachment Techniques and Technical Mistakes Made During Tick Removal,” Ulusal Travma ve Acil Cerrahi Dergisi 26, no. 3 (2020): 405–410, 10.14744/tjtes.2020.59680. 32436976

[10] S. Roupakias , P. Mitsakou , and A. A. Al Nimer , “Surgical Tick Removal,” Wilderness & Environmental Medicine 23, no. 1 (2012): 97–99, 10.1016/j.wem.2011.09.003. 22137906

[11] K. Miyamoto and Y. Hashimoto , “Prevention of Lyme Borreliosis Infection After Tick Bites,” Kansenshōgaku Zasshi 72, no. 5 (1998): 512–516, 10.11150/kansenshogakuzasshi1970.72.512. 9642941

[12] E. Dervis and A. A. Akin Belli , “Benefits of Dermoscopy for Skin Lesions Confusing With Tick Bite and Tick Bites Confusing With Skin Lesions,” Journal of the European Academy of Dermatology and Venereology 30, no. 4 (2016): 715–716, 10.1111/jdv.13011. 25678164

[13] M. Matsuda , N. Oiso , Y. Yano , and A. Kawada , “Dermoscopy for Tick Bite: Reconfirmation of the Usefulness for the Initial Diagnosis,” Case Reports in Dermatology 3, no. 1 (2011): 94–97, 10.1159/000328181. 21577370 PMC3094682

[14] S. Murasawa and T. Kimura , “Histopathological Analysis of 62 Cases of Tick Bite With Tick,” Japanese Journal of Dermatology 115, no. 4 (2005): 571–578, 10.14924/dermatol.115.571.

